# Molecular Identification and Pathogenicity of *Diaporthe eres* and *D. hongkongensis* (Diaporthales, Ascomycota) Associated with Cherry Trunk Diseases in China

**DOI:** 10.3390/microorganisms11102400

**Published:** 2023-09-26

**Authors:** Pengzhao Chen, Pranami D. Abeywickrama, Shuxian Ji, Yueyan Zhou, Xinghong Li, Wei Zhang, Jiye Yan

**Affiliations:** 1 Beijing Key Laboratory of Environment-Friendly Management on Fruit Diseases and Pests in North China, Institute of Plant Protection, Beijing Academy of Agriculture and Forestry Sciences, Beijing 100097, China; pengzhao9609@163.com (P.C.); pranamiabeywickrama@yahoo.com (P.D.A.); jishuxiansdau@163.com (S.J.); 18831176823@163.com (Y.Z.); lixinghong1962@163.com (X.L.); yanjiye@baafs.net.cn (J.Y.); 2 Center of Excellence in Fungal Research, Mae Fah Luang University, Chiang Rai 57100, Thailand; 3 School of Science, Mae Fah Luang University, Chiang Rai 57100, Thailand

**Keywords:** Diaporthaceae, trunk disease, Koch’s postulates, new record, phylogeny, *Prunus*, stone fruits

## Abstract

This study aimed to identify fungal species associated with trunk diseases of sweet cherries (*Prunus avium*) in several commercial cherry orchards in Beijing, Guizhou and Shandong provinces, China. In total, eighteen fungal strains that fitted well into the species concept of *Diaporthe* were isolated. Based on both morphological and multi-locus phylogenetic analyses of internal transcribed spacer region (ITS), beta-tubulin (*tub-2*), calmodulin (*Cal*) and translation elongation factor 1-α (*tef1–α*) sequencing data, fourteen isolates were identified as *Diaporthe eres*, while four isolates were classified as *D. hongkongensis*. Here, we report *D. hongkongensis* causing sweet cherry branch dieback disease and, further, we confirmed the host association of *D. eres* with sweet cherries in China. A pathogenicity assay revealed the ability of both *D. eres* and *D. hongkongensis* to cause shoot necrosis and stem lesions on *Prunus avium* cv. ‘Brooks’ (mean lesion lengths of 1.86 cm and 1.56 cm, respectively). The optimal temperature for the growth of both *Diaporthe* species was tested. The optimal growth temperature for *D. hongkongensis* was 30 °C, and the 25–28 °C temperatures were the most favorable for the growth of *D. eres* strains. This research advances the understanding of fungal trunk diseases in fruit crops, particularly gummosis and branch dieback disease in Chinese cherry orchards, and will aid growers in making decisions about cultural practices and disease management.

## 1. Introduction

Cherries belong to *Prunus* in the Rosaceae, and they are widely cultivated for their aromatic, bright-colored and tasty fruits [1,2]. Many *Prunus* species are grown worldwide, including black cherry (*Prunus serotine*), sour cherry/tart or pie cherry (*Prunus cerasus*), sweet cherry (*Prunus avium*) and West Indian cherry (*Prunus myrtifolia*) [2].

China is one of the largest cherry producers in the world. In 2018, a 163 kha planted area produced approximately 1.35 million tonnes of fruits in China [1,3,4,5]. Sweet cherries ripen earlier than other deciduous fruit trees that are planted in open fields in Northern China, and farmers can often obtain a high price for their production [3,4,5]. However, severe yield losses can occur due to many abiotic and biotic factors [1,6].

Plantation crops, especially pome fruits, stone fruits, nut crops, grapevines, citrus and olive, are threatened by pathogenic fungi [6,7,8,9,10]. Fungal trunk diseases are a significant problem in fruit crops because they can cause devastating tree damage, reduce fruit production and reduce the lifespan of the hosts [7,11]. These diseases primarily affect the trunk and branches of trees, frequently resulting in wood degradation, decreased vigor and, eventually, tree mortality [8,11]. Several fungal species, especially species of Botryosphaeriaceae, Diaporthaceae and Diatrypaceae, have been identified as fungal trunk disease pathogens [8,10,11]. Several Basidiomycota fungi have also been reported to cause fungal trunk diseases [11]. The majority of these fungi infect host wood, mostly through wounds and the subsequent colonization of vascular tissues. After entering through the wounds or natural openings, some of these fungi live as an endophyte and spread through asymptomatic plant materials. This process may increase concerns about the quarantine regulations of a particular country or region [11,12].

Abiotic stresses are also strongly involved, especially in fungal trunk diseases [11], and abiotic stresses can have a synergistic effect in fungal trunk diseases by compromising plant defenses and creating conditions favorable to fungal infection [11]. Understanding these interactions is also crucial for developing effective strategies to manage both abiotic stresses and fungal diseases in plants.

Cherry gummosis and branch dieback disease are two important diseases affecting sweet cherries worldwide [4,6], and due to the complexity of their etiologies, controlling these trunk diseases is difficult [11]. This study was carried out to identify and characterize the pathogens involved in cherry gummosis and branch dieback disease in Chinese cherry orchards.

## 2. Materials and Methods

### 2.1. Field Surveys and Isolation of Fungi

**Figure 1:**
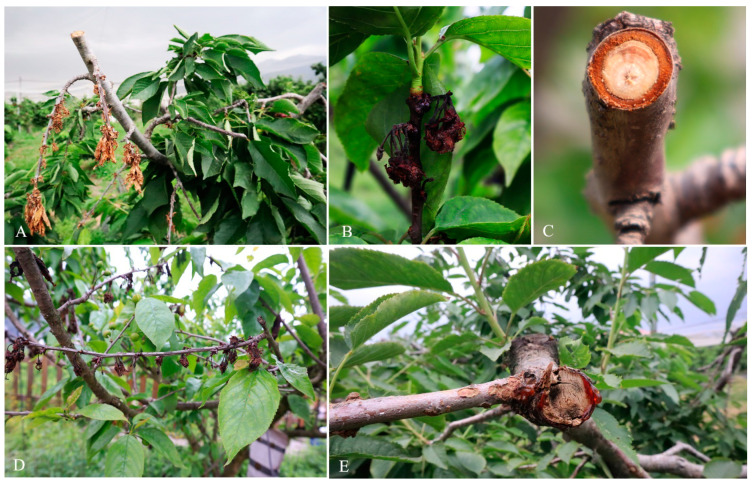
Field symptoms of sweet cherry branch dieback (**A**,**B**,**D**) and gummosis (**E**) and the cross-section of a diseased cherry branch (**C**).

In the survey of fungal trunk disease pathogens in Chinese fruit crops conducted in April 2021, ten symptomatic diseased cherry branches with typical gummosis and branch dieback diseases were collected from three localities in China (Beijing, Guizhou and Shandong) (Figure 1). Samples were taken to the laboratory for further observations. Diseased branches were washed with water and air-dried. Tissue isolations were carried out as described in [13,14]. Tissue pieces (each about 5 mm^2^) were cut from the edge of the healthy and diseased areas that exhibited gummosis and dieback symptoms. Tissues were then surface sterilized using 1.5% NaOCl (sodium hypochlorite) for 1 min. Later, tissues were immersed in 75% ethyl alcohol for 1 min., and washed three times in sterilized distilled water. Then, the tissues were air-dried and placed on PDA plates (Potato dextrose agar). PDA plates were incubated at room temperature. Typical fungal colonies grown from plant tissue pieces were sub-cultured onto new PDA plates. Single hyphal tip isolation was carried out to obtain pure cultures, as illustrated in [13]. Newly sub-cultured PDA plates were observed under the microscope to reveal the septate hyphae and the hyphal tip was isolated using a sterilized needle and/or toothpick [15]. Purified cultures were saved at 4 °C.

All fungal isolates were deposited in the culture collection of the Beijing Academy of Agriculture and Forestry Sciences (JZB). Dry cultures were deposited as herbarium materials in the Herbarium at the Beijing Academy of Agriculture and Forestry Sciences (JZBH).

### 2.2. Molecular Identification of Fungi

#### 2.2.1. Fungal DNA Sequencing

Total genomic DNA was extracted from 5–7-day-old pure cultures using the TIANcombi DNA Lyse and Det PCR Kit (Tiangen Biotech Co., Ltd., Beijing, China) following the manufacturer’s protocols. In total, four gene regions were sequenced and amplified (Table 1). The PCR mixture for all gene regions was as follows: 1 μL of genomic DNA, 45 μL of Golden Star T6 Super PCR mix (1.1×) (Tsingke Biotechnology Co., Ltd., Beijing, China), 2 μL of 10 μM of each forward and reverse primer up to a total volume of 50 μL. All gene regions with respective primer pairs and annealing temperatures are given in Table 1. The PCR was performed in a C1000 TouchTM thermal cycler (Bio-Rad Laboratories Inc., Hercules, CA, USA), and the results were observed on 1.5% agarose gel electrophoresis under ultraviolet light using GelDocXR+ (Bio-Rad Laboratories Inc., Hercules, CA, USA). All of the PCR products were sequenced by a commercial sequence provider (Sinogenomax Co., Ltd., Beijing, China).

#### 2.2.2. Phylogenetic Analysis

Sequences generated in this study were checked for sequencing quality by checking chromatograms of sequences using BioEdit v.7.0.9.0. All sequences generated in this study are deposited in NCBI GenBank (Table 2).

The initial species were identified via the search against the sequences in the NCBI BLASTn (https://blast.ncbi.nlm.nih.gov/Blast.cgi, accessed on 4 April 2023), and relevant sequences were downloaded from NCBI following recent taxonomic treatments [21,22]. Sequences were aligned using MAFFT v. 7 (http://mafft.cbrc.jp/alignment/server, accessed on 4 April 2023). Combined and single gene files were prepared and analyzed based on each genus/family, as given in previous studies. Phylogenetic analysis was inferred via maximum likelihood (ML) and Bayesian analysis (BI).

The evolutionary models for Bayesian and ML analyses were selected using jModelTest v. 2.0 [23,24]. Both ML and BI analyses were carried out using the CIPRES Science Gateway portal (www.phylo.org, accessed on 4 April 2023) [25]. Maximum likelihood (ML) analyses were conducted using RaxML-HPC2 on XSEDE v. 8.2.8 [26]. The GTR + I + G evolution model was used. Bayesian Inference (BI) analyses were performed using MrBayes v. 3.2.7 [27]. The Markov chain Monte Carlo sampling (BMCMC) analysis was conducted with six simultaneous Markov chains. The MCMC heated chain was set at a 05 “temperature” value and run for 1,000,000 generations. Trees were sampled at every 100th generation. From the 10,000 trees obtained, the first 2000 trees representing the burn-in phase were discarded. To calculate posterior probabilities, the remaining 8000 trees were used.

### 2.3. Morphological Characterization

Fungal colonies grown on PDA or PNA (pine needles + water agar) at 25 °C, and after seven days, they were used to examine the morphological characters. Colony morphology and conidial characteristics were examined. Images of morphological structures were taken using an Axio Imager Z2 photographic microscope (Carl Zeiss Microscopy, Oberkochen, Germany). Measurements were taken using ZEN PRO 2012 (Carl Zeiss Microscopy, Oberkochen, Germany). The conidial size was measured for each isolate, and the mean values were calculated.

### 2.4. Temperature Growth Studies

Two isolates from each species (*Diaporthe eres*: JZB320215, JZB320216 and *D. hongkongensis*: JZB320202, JZB320204) were tested to identify their optimal growth temperature conditions. One PDA plate with each isolate was incubated for 7 days at 25 °C. From these cultures, a 5-millimeter-wide plug was taken and placed in the center of the new PDA plates. Three replicate plates per isolate were incubated at 5, 10, 15, 20, 25, 28, 30 and 35 °C in the dark for three days.

### 2.5. Pathogenicity Study

One-year-old healthy shoots of sweet cherry cv. “Brooks”, about 30 cm long, were inoculated with two representative isolates (*Diaporthe hongkongensis*: JZB320204 and *Diaporthe eres*: JZB320215). In total, eighteen shoots per isolate were used. The shoots were initially washed with running tap water to remove the debris. Then, shoots were wiped with 75% ethanol and immersed in 1% NaOCl for 1 min and washed with sterilized distilled water for 1 min. Then, they were air-dried.

Wounds were made on each shoot using a cork borer (5 mm). Agar plugs with mycelium were placed on the wounds. Then, the wounds were sealed. Inoculated shoots were kept at 25 °C with a 12 h photoperiod. The shoots were covered to maintain a moist environment. Uncolonized PDA plugs were used as a negative control. Pots were arranged in a completely randomized design. The experiment was repeated once.

Five days after inoculation, the disease incidences were calculated (dividing the number of shoots showed the disease symptoms by the total number of shoots used to inoculated in each treatment), and the lesion lengths were measured. After the lesion measurements, shoots were subjected to surface sterilization and re-isolation. All fungal colonies resembling to *Diaporthe* were subjected to morpho-molecular identification to fulfill the Koch’s postulates. The mean lesion length (cm) was calculated, and ANOVA assumptions were verified. The analyses were performed using stats packages SPSS Statistics ver. 25 (IBM).

## 3. Results

During the survey of fungal trunk diseases causing pathogens on fruit crops in China, symptomatic sweet cherry branches were collected in several orchards in Beijing, Guizhou and Shandong provinces. As a result of mycological study of plant tissues, eighteen fungal isolates were isolated.

### 3.1. Molecular Identification and Phylogenetic Analysis

**Figure 2:**
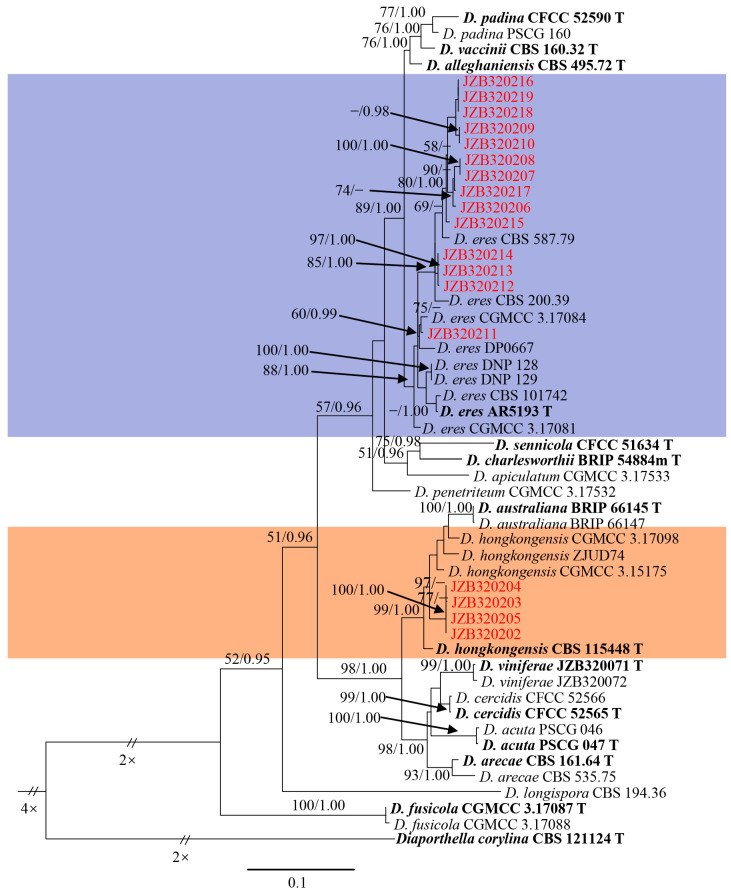
Phylogenetic tree generated via Maximum Likelihood-based combined ITS, *tef1-α*, *Cal* and *tub-2* sequence alignments of *Diaporthe*. *Diaporthella corylina* (CBS 121124) was used as an outgroup taxon. The separate sequence lengths were 510 bp (ITS), 378 bp (*tef1-α*), 386 bp (*Cal*) and 432 bp (*tub-2*). Maximum Likelihood (ML) bootstrap support value (BS ≥ 50) and Bayesian Inference (BI) posterior probabilities (PP ≥ 0.95) were shown on each node. The type strains were indicated in ‘T’ and bold. The isolates in this study were marked in red.

All fungal isolates were sequenced, and the combined ITS, *tef1-α*, *Cal* and *tub-2* sequence data were analyzed based on Maximum Likelihood (ML) and Bayesian Inference (BI). Fourteen isolates were clustered with several reference strains of *D. eres*, including AR5193 (ex-type), and the ML bootstrap support value (BS) and BI posterior probabilities (PP) were 88 and 1.00, respectively (Figure 2). Four isolates were clustered with *D. hongkongensis* (CBS 115448, ex-type; ZJUD74; CGMCC 3.15175; CGMCC 3.17098) with a 99 ML-BS and 1.00 BI-PP value (Figure 2).

The combined gene analyses comprising 1716 characters with gaps and the best RAxML tree with a final likelihood value of -10633.567676 are presented. The matrix had 694 distinct alignment patterns, with undetermined characters being 20.59%. Estimated base frequencies were as follows: A = 0.220258, C = 0.317917, G = 0.232470 and T = 0.229355; substitution rates AC = 1.203612, AG = 2.754249, AT = 1.180540, CG = 0.839140, CT = 4.894412 and GT = 1.000000; gamma distribution shape parameter α = 0.652442.

### 3.2. Morphological Characterization

**Figure 3:**
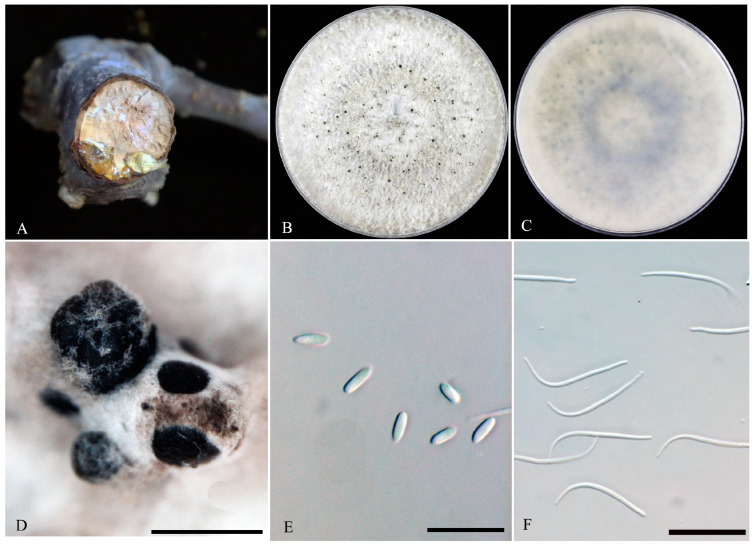
***Diaporthe eres*** (JZB320215). (**A**) gummosis symptom of the sweet cherry branch. Surface (**B**) and reverse (**C**) colony on PDA at 25 °C after seven days. (**D**) Pycnidia on PDA, (**E**) alpha conidia and (**F**) beta conidia. Scale bars: (**D**) = 500 μm, (**E**,**F**) = 20 μm.

**Figure 4:**
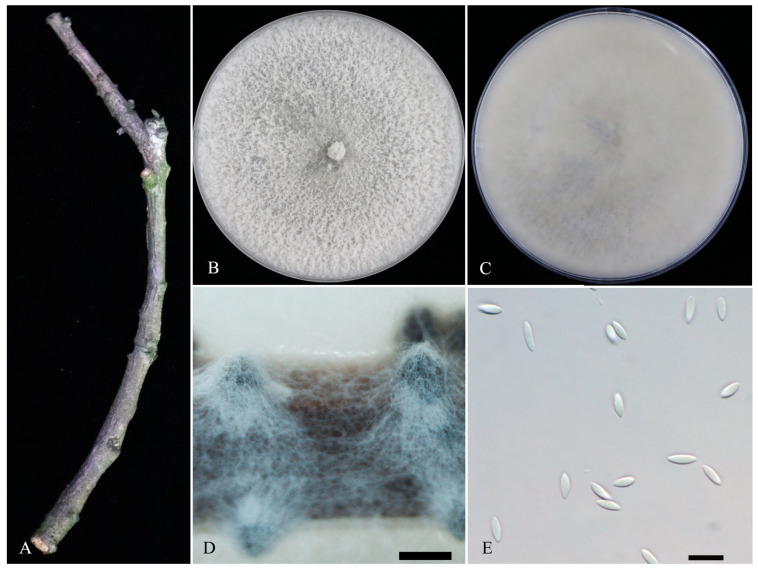
***Diaporthe hongkongensis*** (JZB320204). (**A**) dieback symptom of sweet cherry branch. Surface (**B**) and reverse (**C**) colony on PDA at 25 °C after seven days. (**D**) conidiomata on PNA. (**E**) *alpha* conidia. Scale bars: (**D**) = 500 μm, (**E**) = 10 μm.

Based on the phylogenetic analysis, all fungal isolates were identified as *Diaporthe*. All isolates showed two different morphological characteristics. A total of fourteen isolates of *Diaporthe eres* and four isolates of *D. hongkongensis* were obtained (Figure 3 and Figure 4). The morphological characteristics of JZB320204 (*D. hongkongensis*) and JZB320215 (*D. eres*) were further observed, and the two isolates were comparatively similar in terms of their alpha conidia size, shapes and growth rates. While JZB320204 showed relatively larger alpha conidia (L/W ratio = 2.93) than JZB320215 (L/W ratio = 2.64), the reverse view of the colony was white in JZB320204 and pale black in JZB320215.

### 3.3. Taxonomy

**Diaporthe eres** Nitschke (1870) (Figure 3).

Index Fungorum no: IF 172054.

**Sexual morph**: Not observed. **Asexual morph**: Conidiomata pycnidial, black, formed on PDA, superficial, solitary or aggregated. Conidiophores lining the inner cavity are hyaline and reduced to conidiogenous cells. Conidiogenous cells are hyaline and smooth. Alpha conidia hyaline, ellipsoid, fusiform, aseptate, 5–8.5 × 2–3 μm (mean ± SD = 6.9 ± 0.8 × 2.6 ± 0.3 μm, *n* = 40, L/W ratio = 2.64). Beta conidia hyaline, aseptate, filiform, 22–31 × 1–1.5 μm (mean ± SD = 27.7 ± 2 × 1.3 ± 0.1 μm, *n* = 40, L/W ratio = 21.19). Gamma conidia were not observed.

**Culture characteristics**: Colonies were flat, with sparse-to-moderate aerial mycelium, growing on a PDA plate reaches 60 mm after 2 days at 25 °C in 12 h light/dark, with a 27 mm/d growth rate. On PDA, pale grey to grey and reverse pale grey–smoke grey.

**Material examined**: China, Beijing, Tongzhou district, from diseased branches of *Prunus avium* with gummosis symptoms, 9 April 2021, P. Chen, dried culture: JZBH320215, living culture: JZB320215 = TZZG36-S1. Other isolates studied are listed in Table 2.

**Notes**: *Diaporthe eres* have been identified as a plant pathogen causing leaf spots and stem cankers in many economically important plant hosts [28,29]. This species was first identified from the twigs of *Ulmus* sp. in Germany and has a worldwide distribution in both temperate and tropical regions [28]. *Diaporthe eres* is listed as a pathogen with plant health inspection and quarantine significance [28,30]. We were able to isolate fourteen strains of *D. eres* (JZB320206–JZB320219), which clade within the *Diaporthe eres* species complex. All our *D. eres* isolates share similar morphology (black pycnidial conidomata, hyaline conidiophores and conidiogenous cells, ellipsoidal and aseptate alpha conidia) with the ex-epitype strain (AR5193), with minor dimensional differences [28,29]. These dimensional differences are probably due to variation in the host associations or the different culture media. The comparison of ITS, *Cal*, *tef1-α* and *tub-2* regions of our *D. eres* isolates with ex-epitype strain (AR5193) reveals 81–89%, 73–76%, 88–92% and 87–93% base pair similarities, respectively.

**Diaporthe hongkongensis** R.R. Gomes, Glienke and Crous (2013) (Figure 4).

Index Fungorum no: IF 802934.

**Sexual morph**: Not observed. **Asexual morph**: Conidiomata pycnidial, black, formed on PNA after 20 days, superficial, solitary or aggregated. Conidiophores lining the inner cavity are hyaline and reduced to conidiogenous cells. Conidiogenous cells are hyaline and smooth. Alpha conidia fusiform, aseptate, 6–8.5 × 2–2.9 μm (mean ± SD = 7 ± 0.6 × 2.5 ± 0.1 μm, *n* = 40, L/W ratio = 2.93). Beta and gamma conidia were not observed.

**Culture characteristics**: Colonies growing on PDA reached 57–60 mm after 2 days in 12 h light/dark, with a 27 mm/d growth rate; after 7 days, white and reverse grey–white to white.

**Material examined**: China, Guizhou Province, Guiyang city, from diseased branches of *Prunus avium* with dieback symptoms, 9 April 2021, P. Chen, dried culture: JZBH320204, living culture: JZB320204 = GZZG2-S3. Other isolates studied are listed in Table 2.

**Note**: *Diaporthe hongkongensis* was initially reported from *Dichroa febrifuga* from Hong Kong as *Phomopsis pittospori* [31]. Later, this species was identified from *Camelia sinensis*, *Citrus* sp. and *Vitis vinifera* [32]. This species was reported as causing stem-end rot on kiwifruit [33], shoot blight and leaf spot on kiwifruit [34] and fruit rot disease on peach [35]. Further, this species was reported as causing top blight in *Cunninghamia lanceolata* [36] and shoot canker in pear [37]. *Diaporthe hongkongensis* belongs to *Diaporthe arecae* species complex [21,22], and in this study, we have recovered four isolates from sweet cherries in China. All four isolates share similar morphologies (superficial pycnidial conidiomata and hyaline, fusiform alpha conidia) to that of the ex-type strain of *D. hongkongensis* (CBS 115448), with minor dimensional differences [22,31]. Comparison of ITS, *Cal* and *tef1-α* and *tub-2* regions of our *D. hongkongensis* isolates with ex-type strain (CBS 115448) reveals 87–91%, 64%, 86–88% and 90–92% base pair similarities, respectively.

### 3.4. Pathogenicity Study

**Figure 5:**
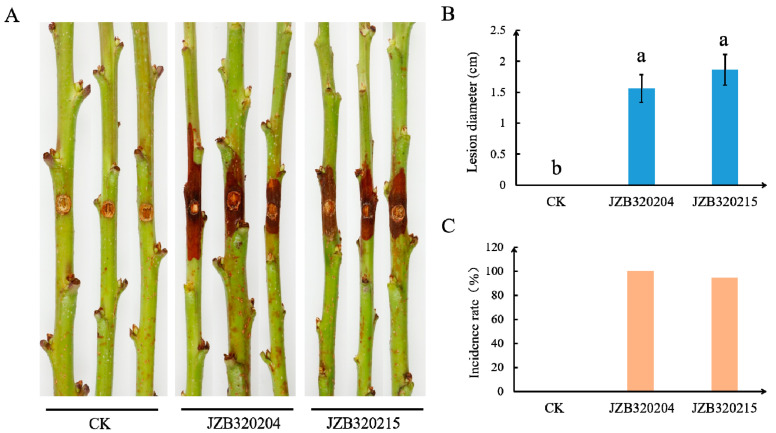
Pathogenicity of *Diaporthe hongkongensis* (JZB320204) and *D. eres* (JZB320215) isolates to *Prunus avium* cv. ‘Brooks’. (**A**) Control (CK) and the representative symptoms on one-year-old detached branches (5 dpi). (**B**) Mean lesion lengths on shoots inoculated in vitro after 5 d (bars indicates the standard errors (*p* < 0.05)). (**C**) Disease incidences of shoots inoculated in vitro.

After five days of inoculation on wounded one-year-old detached shoots of sweet cherry (cv. ‘Brooks’), both species, namely *Diaporthe hongkongensis* (JZB320204) and *D. eres* (JZB320215), caused shoot browning and necrosis (Figure 5A). None of the control shoots showed any disease or symptoms (Figure 5A,C). *Diaporthe hongkongensis* (JZB320204) caused symptoms on all inoculated shoots (with disease incidence of 100%), and the isolate of *D. eres* (JZB320215) caused symptoms on 94.4% of the shoots. The average length of lesions was significantly larger than that of the control, reaching 1.56 cm and 1.86 cm, respectively (Figure 5B). Re-isolated fungi share similar morphological characteristics to primary inoculated isolates, and this observation was further confirmed via the sequencing of the ITS region.

### 3.5. Temperature Growth Studies

**Figure 6:**
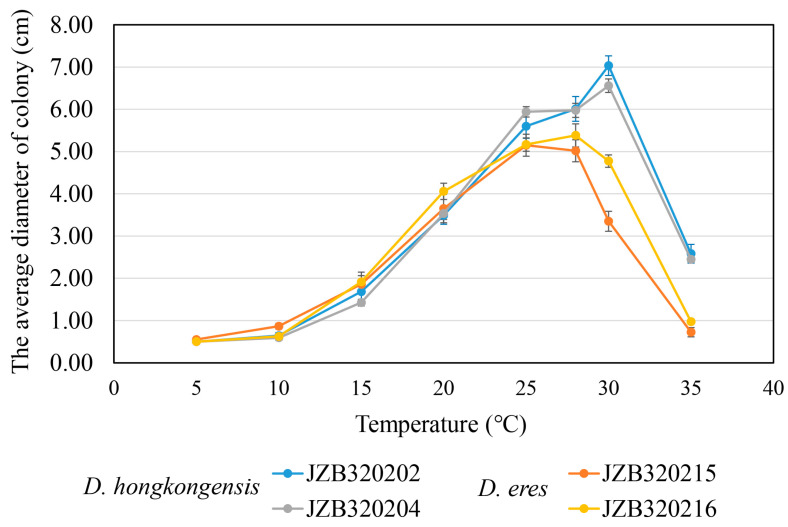
The average diameter of *D. hongkongensis* and *D. eres* cultured under different temperatures (3 dpi). The bar indicates the standard deviation (SD) (*n* = 3).

Two isolates derived from each species were used to test for the optimal growth temperature (*Diaporthe eres*: JZB320215, JZB320216 and *D. hongkongensis*: JZB320202, JZB320204), and the results are shown in Figure 6. The optimal growth temperature of *D. hongkongensis* was 30 °C, and the optimal growth temperatures of the two *D. eres* strains were 25 °C (JZB320215) and 28 °C (JZB320216), respectively. However, both *Diaporthe hongkongensis* and *D. eres* did not grow at 5 °C. During 5–20 °C temperatures, the colony size of both *D. hongkongensis* and *D. eres* is similar. At the temperature of 25–35 °C, the colony diameter of *D. hongkongensis* is significantly higher than that of *D. eres*, indicating that *D. hongkongensis* is more resistant to high temperatures than *D. eres*.

## 4. Discussion

During our survey, the branch samples of *Prunus avium* that showed dieback and gummosis disease symptoms were collected from three locations in China. The surveyed orchards situated in Beijing and Shandong shared similar climatic conditions (temperate monsoon climate), while orchards in Guizhou belonged to the humid subtropical monsoon climate. The incidence of dieback disease in Beijing was 10%, while orchards in Shandong showed a 15–25% incidence rate. A total of eighteen fungal isolates were obtained, and all fungal isolates fitted well with the species concept of *Diaporthe* [21,22]. Phylogenetic analyses based on the four combined loci (ITS, *tef1-α*, *Cal* and *tub-2*) coupled with morphology revealed two *Diaporthe* species (viz. *D. eres* and *D. hongkongensis*) associated with sweet cherry die-back and gummosis disease in China. We do not observe the mixed fungal infection in the same branch sample. But we observe the two-disease occurring on the same orchard. In most cases, *Diaporthe hongkongensis* was isolated from dieback samples, and *D. eres* was isolated from both dieback and gummosis samples.

*Diaporthe* (Diaporthales, Pezizomycotina, Ascomycota) species are endophytic, saprophytic or parasitic, being widely distributed throughout the world and reported in different host plants [21,22,29,38,39]. Many pathogenic *Diaporthe* species cause diseases in many important crops, including grape (*Vitis vinifera*) [40], pear (*Pyrus pyrifolia*) [37], apple (*Malus domestica*) [41], blueberry (*Vaccinium corymbosum*) [42] and *Toxicodendron vernicifluum* [43], and cause symptoms such as dieback, canker, rot and leaf spots [44]. *Diaporthe hongkongensis* and *D. eres* have also been reported to cause diseases in woody plants, especially causing branch dieback and canker. *Diaporthe hongkongensis* identified on *Actinidia deliciosa* (Kiwifruit) [33], *Castanopsis eyrei* [45], *Citrus sinensis* [46], *Cyclobalanopsis glauca* [45], *Dichroa febrifuga* [31], *Herba Patriniae* [45], *Llex Latifolia* [45], *Loropetalum chinensis* [45], *Miscanthus sinensis* [45], *Smilax china* [45], *Ternstroemia gymnanthera* [45], *Vitis vinifera* [32] and *D. eres* reported a wide variety of host plants [21,22,29].

The Rosaceae family comprises a large number of plant species, and it extends from herbaceous and ornamental plants to fruit trees [47]. Most of the species in Rosaceae are regarded as economically important crops, such as strawberry, apple, cherry, almond, peach, plum, pear, blackberry and raspberry [47]. Even though *D. eres* is a well-known fungus, there are few studies of their pathogenicity on the above-mentioned hosts [47]. However, this species is identified as a pathogen and cause of canker and shoot blight in peaches, cane blight in blackberry, trunk canker and death in apple trees and wilting of shoots in *Cotoneaster* species [47]. In China, *D. perniciosa* (as *Phomopsis perniciosa*) has previously been reported to cause cherry stem canker disease in plants in Shandong Peninsula [48]. They have observed symptoms of canker and branch dieback, including cracks, black pycnidia and cirri containing α-conidia [48]. However, according to [21] and [22], based on their ITS phylogeny, *D. perniciosa* has been grouped with *D. asheicola* (CBS 136967) and other reference isolates of *D. eres*. Therefore, we also suspected that *D. perniciosa* is a species of *D. eres*, and through this study, we confirmed its host association and the pathogenicity of sweet cherries in China. Interestingly, in this study, we isolated *D. eres* from symptomatic branches of both dieback and gummosis disease. However, we were unable to observe the gummosis symptoms when we artificially inoculated one-year-old cherry shoots during the pathogenicity experiments. Several other pathogens have also been reported to cause gummosis disease in sweet cherries, including *Botyosphaeria dothidea* [49], *Valsa mali* [50], *Pseudomonas syringae* pv. *syringae* [51] and P. *syringae* pv. *morsprunorum* R1 (Race 1) and R2 (Race 2). Further, this study is the first study to report that *D. hongkongensis* is associated with sweet cherries and can cause branch dieback in cherries.

A study on the temperature range of fungal growth showed that the optimal temperature for *D. hongkongensis* is 30 °C, and for *D. eres* it is 25–28 °C. Proper cultural practices, such as maintaining optimal irrigation, managing proper spacing, nutrient management and providing protective measures during extreme weather events, can help to mitigate the impacts of abiotic stresses on plant health and reduce susceptibility to fungal trunk diseases [11]. According to [52], while almond production has been expanding in Spain, the incidence of fungal diseases is increasing and compromises crop productivity. According to the authors, the prevalence of shoot cankers and blight caused by *Diaporthe* species is higher in coastal areas with higher humidity and milder temperatures [51]. Even though the cherries are often planted in open fields where temperature control is challenging, understanding the temperature requirements of fungal pathogens will helps farmers to check for signs of disease or the development and progression of fungal diseases.

## 5. Conclusions

This study represents the investigation of 18 fungal isolates of *Diaporthe* species causing on gummosis and branch dieback diseases of sweet cherries in China. According to molecular and morphological data fourteen isolates were identified as *Diaporthe eres* and other four as *D. hongkongensis*. Our result demonstrated that both *D. eres* and *D. hongkongensis* are pathogenic to sweet cherry shoots, but the optimal growth temperatures for these fungi differ. Further studies are necessary to understand the factors that determine the pathogenicity of these *Diaporthe* strains towards fruit trees, especially stone fruits. An extensive collection of disease samples of sweet cherries with expanded investigations throughout China is also recommended.

## Figures and Tables

**Table 1:** Gene regions and primers used in this study.

Gene Region	Primer Pairs	Sequence (5′—3′)	Reference
ITS	ITS1 ITS4	TCCGTAGGTGAACCTGCGG TCCTCCGCTTATTGATATGC	[16]
*Tef1-α*	EF1-688F EF1-1251R EF1-728F EF1-986R	CGGTCACTTGATCTACAAGTGC CCTCGAACTCACCAGTACCG CATCGAGAAGTTCGAGAAGG TACTTGAAGGAACCCTTACC	[17,18]
*tub-2*	Bt2a Bt2b TUB2Fd TUB4Rd	GGTAACCAAATCGGTGCTGCTTTC ACCCTCAGTGTAGTGACCCTTGGC GTBCACCTYCARACCGGYCARTG CCRGAYTGRCCRAARACRAAGTTGTC	[19,20]
*Cal*	CAL-228F CAL-737R	GAGTTCAAGGAGGCCTTCTCCC CATCTTTCTGGCCATCATGG	[18]

**Table 2:** Strains/isolates and GenBank numbers used in this study.

Species	Culture No.	Origin	GenBank Number			
			ITS	*Cal*	*tef1-α*	*tub-2*
*D. acuta*	PSCG 046	China	MK626958	MK691124	MK654803	MK691224
*D. acuta*	PSCG 047 *	China	MK626957	MK691125	MK654802	MK691225
*D. alleghaniensis*	CBS 495.72 = ATCC 24097 *	Canada	KC343007	KC343249	KC343733	KC343975
*D. apiculatum*	CGMCC 3.17533	China	KP267896	-	KP267970	KP293476
*D. arecae*	CBS 161.64 *	India	KC343032	KC343274	KC343758	KC344000
*D. arecae*	CBS 535.75	Suriname	KC343033	KC343275	KC343759	KC344001
*D. australiana*	BRIP 66145 *	Australia	MN708222	-	MN696522	MN696530
*D. australiana*	BRIP 66147	Australia	MN708224	-	MN696523	MN696532
*D. cercidis*	CFCC 52565*	China	MH121500	MH121424	MH121542	MH121582
*D. cercidis*	CFCC 52566	China	MH121501	MH121425	MH121543	MH121583
*D. charlesworthii*	BRIP 54884 m *	Australia	KJ197288	-	KJ197250	KJ197268
*D. eres* *D. eres*	AR5193 * CBS 101742	Germany Netherlands	KJ210529 KC343073	KJ434999 KC343315	KJ210550 KC343799	KJ420799 KC344041
*D. eres (=D. biguttusis)*	CGMCC 3.17081 *	China	KF576282	-	KF576257	KF576306
*D. eres (=D. castaneae-mollisimae)*	DNP 128 *	China	JF957786	JX197430	JX275401	JX275438
*D. eres (=D. castaneae-mollisimae)*	DNP 129	China	JQ619886	JX197431	JX275402	JX275439
*D. eres (=D. cotoneastri)*	DP0667	-	KC843328	KC843155	KC843121	KC843229
*D. eres (=D. ellipicola)*	CGMCC 3.17084 *	-	KF576270	-	KF576245	KF576294
*D. eres (=D. nobilis)*	CBS 200.39	-	KC343151	KC343393	KC343877	KC344119
*D. eres (=D. nobilis)*	CBS 587.79	-	KC343153	KC343395	KC343879	KC344121
***D. eres***	**JZB320206**	**Guizhou, China**	**OM980309**	**OQ473424**	**OQ513364**	**ON152804**
***D. eres***	**JZB320207**	**Guizhou, China**	**OM980310**	**OQ473425**	**OQ513365**	**ON152805**
***D. eres***	**JZB320208**	**Guizhou, China**	**OM980311**	**OQ473426**	**OQ513366**	**ON152806**
***D. eres***	**JZB320209**	**Guizhou, China**	**OM980312**	**OQ473427**	**-**	**ON152807**
***D. eres***	**JZB320210**	**Guizhou, China**	**OM980313**	**OQ473428**	**-**	**ON152808**
***D. eres***	**JZB320211**	**Beijing, China**	**OM980314**	**OQ473429**	**OQ513367**	**ON152809**
***D. eres***	**JZB320212**	**Beijing, China**	**OM980315**	**OQ473430**	**OQ513368**	**ON152810**
***D. eres***	**JZB320213**	**Beijing, China**	**OM980316**	**OQ473431**	**OQ513369**	**ON152811**
***D. eres***	**JZB320214**	**Beijing, China**	**OM980317**	**OQ473432**	**OQ513370**	**ON152812**
***D. eres***	**JZB320215**	**Beijing, China**	**OM980318**	**OQ473433**	**OQ513371**	**ON152813**
***D. eres***	**JZB320216**	**Beijing, China**	**OM980319**	**OQ473434**	**OQ513372**	**ON152814**
***D. eres***	**JZB320217**	**Shandong, China**	**OM980320**	**OQ473435**	**OQ513373**	**ON152815**
***D. eres***	**JZB320218**	**Shandong, China**	**OM980321**	**OQ473436**	**OQ513374**	**ON152816**
***D. eres***	**JZB320219**	**Shandong, China**	**OM980322**	**OQ473437**	**OQ513375**	**ON152817**
*D. fusicola*	CGMCC 3.17087 *	China	KF576281	KF576233	KF576256	KF576305
*D. fusicola*	CGMCC 3.17088	China	KF576263	KF576221	KF576238	KF576287
*D. hongkongensis*	CBS 115448 *	China	KC343119	KC343361	KC343845	KC344087
*D. hongkongensis*	ZJUD74	China	KJ490609	-	KJ490488	KJ490430
*D. hongkongensis (=D. lithocarpus)*	CGMCC 3.15175 *	-	KC153104	KF576235	KC153095	KF576311
*D. hongkongensis (=D. lithocarpus)*	CGMCC 3.17098	-	KF576276	KF576228	KF576251	KF576300
***D. hongkongensis***	**JZB320202**	**Guizhou, China**	**OM980305**	**OQ473420**	**OQ513360**	**OL845879**
***D. hongkongensis***	**JZB320203**	**Guizhou, China**	**OM980306**	**OQ473421**	**OQ513361**	**OL845880**
***D. hongkongensis***	**JZB320204**	**Guizhou, China**	**OM980307**	**OQ473422**	**OQ513362**	**OL845881**
***D. hongkongensis***	**JZB320205**	**Guizhou, China**	**OM980308**	**OQ473423**	**OQ513363**	**OL845882**
*D. longispora*	CBS 194.36	-	MH855769	KC343377	KC343861	KC344103
*D. padina*	CFCC 52590 *	China	MH121525	MH121443	MH121567	MH121604
*D. padina*	PSCG 160	-	MK626892	MK691172	MK654851	MK691261
*D. penetriteum*	CGMCC 3.17532	China	KP267879	-	KP267953	KP293459
*D. sennicola*	CFCC 51634 *	China	KY203722	-	KY228883	KY228889
*D. vaccinii*	CBS 160.32 = IFO 32646 *	USA	KC343228	KC343470	KC343954	KC344196
*D. viniferae*	JZB320071 *	Guangxi, China	MK341551	MK500119	MK500107	MK500112
*D. viniferae*	JZB320072	Guangxi, China	MK341552	MK500120	MK500108	MK500113
*Diaporthella corylina*	CBS 121124 *	China	KC343004	KC343246	KC343730	KC343972

AR: Culture collection of Systematic Mycology and Microbiology Laboratory, USDA-ARS, Beltsville, Maryland, USA; BRIP: Queensland Plant Pathology’s herbarium/culture collection, Australia; CBS: Culture collection of the Centraalbureau voor Schimmelcultures, Fungal Biodiversity Centre, Utrecht, The Netherlands; CFCC: China Forestry Culture Collection Center, China; CGMCC: China General Microbiological Culture Collection; JZB: Beijing Academy of Agriculture and Forestry Sciences’ culture collection. ZJUD: Zhejiang University, Hangzhou, China. Isolates obtained from this study are in bold. * = type strain.

## Data Availability

The sequence data obtained in this study are openly available in NCBI GenBank and the accession numbers are stated in the article.
